# Impact of the Organizational Model Adopted during the COVID-19 Pandemic on the Perceived Safety of Intensive Care Unit Staff

**DOI:** 10.3390/jcm11061487

**Published:** 2022-03-09

**Authors:** Elena Conoscenti, Maria Campanella, Antonino Sala, Maria Cristina Di Stefano, Dario Vinci, Rosario Lombardo, Giuseppe Arena, Angelo Ginestra, Rosario Fiolo, Fabio Tuzzolino, Alessia Ippolito, Gennaro Martucci, Giuseppe Enea, Angelo Luca

**Affiliations:** 1 Direzione delle Professioni Sanitarie, IRCCS-ISMETT (Istituto Mediterraneo per I Trapianti E Terapie Ad Alta Specializzazione), Via E. Tricomi 5, 90127 Palermo, Sicily, Italy; econoscenti@ismett.edu (E.C.); mcampanella@ismett.edu (M.C.); rlombardo@ismett.edu (R.L.); garena@ismett.edu (G.A.); aippolito@ismett.edu (A.I.); genea@ismett.edu (G.E.); 2 Direzione Generale, IRCCS-ISMETT (Istituto Mediterraneo per I Trapianti E Terapie Ad Alta Specializzazione), Via E. Tricomi 5, 90127 Palermo, Sicily, Italy; asala@ismett.edu (A.S.); ftuzzolino@ismett.edu (F.T.); aluca@ismett.edu (A.L.); 3 Direzione Sanitaria, IRCCS-ISMETT (Istituto Mediterraneo per I Trapianti E Terapie Ad Alta Specializzazione), Via E. Tricomi 5, 90127 Palermo, Sicily, Italy; mcdistefano@ismett.edu (M.C.D.S.); dvinci@ismett.edu (D.V.); 4 Ospedale Civico, Via C. Lazzaro 2, 90127 Palermo, Sicily, Italy; angeloginestra@gmail.com; 5 Dipartimento BIND, Scuola di Medicina e Chirurgia, Università degli Studi di Palermo, Via del Vespro, 129, 90127 Palermo, Sicily, Italy; rosario.fiolo@unipa.it; 6 Department of Anesthesia and Intensive Care, IRCCS-ISMETT (Istituto Mediterraneo per I Trapianti E Terapie Ad Alta Specializzazione), Via E. Tricomi 5, 90127 Palermo, Sicily, Italy

**Keywords:** COVID-19, intensive care unit, safety management, fear, survey

## Abstract

Background: The SARS-CoV-2 pandemic had a devastating health, social, and economic effect on the population. Organizational, technical and structural operations aimed at protecting staff, outpatients and inpatients were implemented in an Italian hospital with a COVID-19 dedicated intensive care unit. The impact of the organizational model adopted on the perceived safety among staff was evaluated. Methods: Descriptive, structured and voluntary, anonymous, non-funded, self-administered cross-sectional surveys on the impact of the organizational model adopted during COVID-19 on the perceived safety among staff. Results: Response rate to the survey was 67.4% (153 completed surveys). A total of 91 (59%) of respondents had more than three years of ICU experience, while 16 (10%) were employed for less than one year. Group stratification according to profession: 74 nurses (48%); 12 medical-doctors (7%); 11 physiotherapists (7%); 35 nurses-aides (22%); 5 radiology-technicians (3%); 3 housekeeping (1%); 13 other (8%). The organizational model implemented at ISMETT made them feel safe during their workday. A total of 113 (84%) agreed or strongly agreed with the sense of security resulting from the implemented measures. A vast majority of respondents perceived COVID-19 as a dangerous and deadly disease (94%) not only for themselves but even more as vectors towards their families (79%). A total of 55% of staff took isolation measures and moved away from their home by changing personal habits. The organizational model was perceived overall as appropriate (91%) to guarantee their health. Conclusion: The vast majority of respondents perceived the overall model applied during an unexpected, emergency situation as appropriate.

## 1. Introduction

The SARS-CoV-2 pandemic had a devastating health, social, and economic effect on the population and has caused a worldwide tragedy that has left hospitals and communities unprepared [1]. In this emergency, like all community health care facilities, our hospital was forced to implement, in a very short time, a series of organizational, technical, structural operations and policies aimed at protecting its staff, outpatients and inpatients, and set up a dedicated intensive care unit (ICU) area for critical COVID-19 patients.

Taking into consideration that the period from the onset of the pandemic to the opening of the COVID-19 unit was short, the organizational model adopted at the hospital underwent changes over months based on previous medical conditions. During Severe Acute Respiratory Syndrome (SARS), the reported rate of hospital infections among health care providers was up to 60%. Therefore, caregivers were to be considered an at risk category with relative and possible risk of social isolation, a condition that also extended to close social relationships [2].

This high incidence of health risk can obviously result in resistance of the clinical staff towards performing their working tasks given that the risk of exposure applies not only to them but also to their families [2].

A comprehensive systematic review [2] focused on factors that determine resistance mechanisms. The study highlighted an increased risk perception in women/mothers with children at home and the fear of contracting the virus among health care providers reached a threshold of 91%, of which 27% considered the possibility of changing jobs.

Yiwen Koh et al. [2] described the risk perception as closely related to ones’ job profile. Women/mothers with children at home, junior staff and nurses showed greater resistance to performing their working tasks compared to physicians, men and more senior staff.

A key role is therefore played, as clearly emerged, by the strategies adopted by local organizations, which varied considerably, and could be grouped into two basic groups: avoiding patient contact, and compliance with prevention measures. External factors influencing risk perception and personal strategies can be directly influenced by the safety of the worker and therefore by the organizational strategies adopted through tools such as adequate staff training, use of adequate PPE, and forms of incentives and compensation [2,3,4].

The World Health Organization (WHO) has monitored and studied the COVID-19 pandemic and is providing national health organizations with specific tools to monitor the level of knowledge and identify preventive behaviors [5]. The WHO also produced a tool for the study and collection of data on risk assessment in the health care context [6]. The actions that were implemented in terms of organizational structure, training, and improvisation measures, left the staff perceiving a safety risk due to a lack of information/experience [7,8,9].

**Figure 1:**
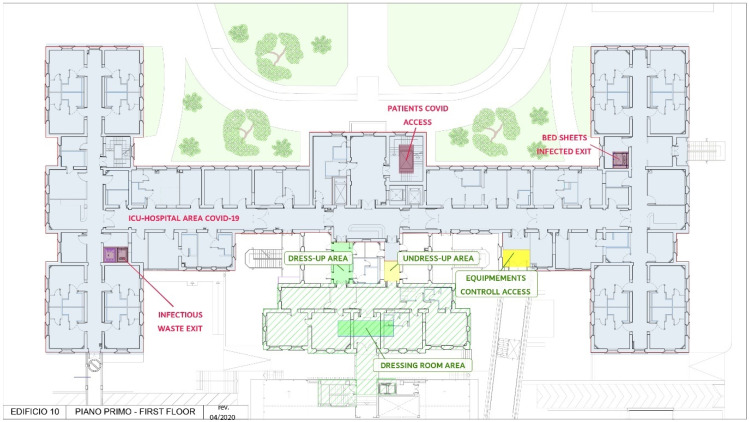
Plant of the unit.

This survey aims to measure how the organizational model adopted by the institution has been perceived as “safe” by the staff working in this sensitive area and how the tools adopted have guaranteed safety in a medical emergency through a survey among the operating staff. (Table 1; Figure 1)

## 2. Materials and Methods

This is a descriptive, structured and voluntary, anonymous, non-funded, self-administered cross-sectional survey conducted at a 107-bed medical institute accredited with Joint Commission International standards and focused on all solid organ transplantations and the care of end-organ failure in which part of the hospital was converted into a COVID-19 ICU.

The Institutional Research Review Board approved the conduction of the survey IRRB/19/21. The need for informed consent was waived given the voluntary nature of the survey and the anonymous data collection. As part of the protection of the health and safety of workers, technical, procedural and organizational measures were implemented based on mandatory legislation and regulations.

Our institution is a regional and extra-regional hub for extracorporeal membrane oxygenation (ECMO) retrieval, nurses are skilled in caring for critically ill patients with acute respiratory distress syndrome [10]. Following the request of the Sicilian Region Health Care Department to open a COVID-19 ICU, training programs were activated for nurses from other units to obtain critical care skills in a short time, in addition to recruiting new nurses. In the early stages, one of the goals of management was to prevent inexperienced nurses from entering the COVID-19 area. Newly hired personnel replaced experienced nurses that were moved to the COVID-19 area.

Furthermore, it was considered of fundamental importance to train all clinical staff on donning and doffing personal protective equipment (PPE) in order to prevent COVID-19 infections [11,12]. About 600 people were trained for a total of about 300 training hours provided. ICU nurses (56) were evaluated by the occupational physician with the release of eligibility opinion for possible assignment to the new COVID-19 unit. Access to the COVID-19 area, dressing, undressing, medical waste management, supplies, patient transfers, and transport of biological samples are only some of the targeted areas for which it was necessary to ensure safety.

Organizational measures and structural changes were put in place by ISMETT to address the COVID-19 emergency and secure patient and staff protection, first by formally introducing policies starting from March 2020, and subsequently with further dedicated policies for the opening of the COVID-19 ICU with the first critical patient admitted on 5 October 2020.

Policies and procedures, regulations, devices, and infrastructures are perceived as adequate forms of personal protection, thus increasing the safety for one’s perceived health [2].

### 2.1. Survey Development, Design, and Conduction

Design of the study—observational study with submission of questionnaires, data collection, statistical analysis with stratification for some characteristics of the studied population.

### 2.2. Primary Outcome

⮚ Perception of safety of staff working in the COVID-19 ICU.

### 2.3. Secondary Outcome

⮚ Incidence of biohazard injuries, perception of training.
⮚ Number of COVID-19+ cases in health care workers in the COVID-19 area found with regular screening after entering the COVID-19 ICU.
⮚ Incidence of tendency to self-isolate in relation to the adopted organizational model.
⮚ Incidence of additional requests for health surveillance interventions in health care personnel working in the COVID-19 area.

The survey was available online on the Survey Monkey System (Survey-Monkey, San Mateo, CA, USA) for one month. The first e-mail was sent on 26 June, with a reminder sent on 25 July. The questionnaire included 27 questions divided into four sections (general data, clinical care activities, preventive measures implemented, staff risk perception).

The survey was developed by five investigators (C.E, C.M, e.g., L.R, A.G.) based on a review of the theoretical basis of survey elaboration, reappraisal of the relevant literature in the field of perception of the infectious risk among health care providers [13,14]. The organizational model adopted at the hospital was analyzed to structure the content of the survey. A panel of senior authors evaluated the content validity of the questionnaire both internally and outside the hospital (A.S., A.G., R.F.) (Table 2).

The questionnaire was administered to the hospital population falling within the inclusion criteria (human resources working in the COVID-19 unit both clinical and non-clinical staff): a total of 227 potential respondents: 207 clinical and 20 non-clinical.

In order to improve the response rate, two reminders emailed. The survey was analyzed individually by the investigators. No major conflicting answers were found, consequently no answers were discarded.

### 2.4. Statistical Analysis

Quantitative and qualitative statistical analyses were used depending on the categorical or continuous nature of the various items making up the questionnaire. Univariate analyses were performed on all items of the questionnaire such as distributions of absolute frequencies with relative frequencies and associative analysis as contingency tables with Fisher’s exact test or Chi–square when appropriate. The analyses were stratified where possible in the following groups:

- Age groups (20–30; 31–40; 41–50; over 50).
- Sex.
- Professional category.
- Years of ICU experience (>6 months; 6 months to 1 year); 1 to 3 years; <3 years).
- Exposure time in the COVID-19 ICU (<1 month; 1 to 3 months; 3 to 6 months; <6 months).
- Family and cohabiting settings.

All analyses were conducted with the aid of SAS 9.4, all statistical tests were two-way and tests with *p* values <0.05 considered significant.

## 3. Results

### 3.1. General Data

A total of 153 completed surveys were obtained out of the 227 requests sent (67.4% of the total potential respondents). A total of fifty six percent (n = 86) were female. Moreover, the respondents were stratified into seven groups according to their profession. The data analysis led us to merge physiotherapists and radiology technicians in one group, and housekeeping and others in another, due to low number of respondents in the individual groups. A total of fifty five respondents (36%) were in the age range of 41–50. There were 91 (59%) respondents with more than three years of ICU experience, while 16 (10%) were employed for less than one year. A total of eighty three (54%) worked in the COVID-19 area between three and six months or more.

A total of one hundred thirteen (84%) agreed or strongly agreed to the sense of security resulting from the implemented measures while nine (6%) considered the implemented measures irrelevant; thirteen (9%) gave a completely negative answer. The acceptance value of the model is lower by nurses with a disagreement that rises to 15% and the perception of safety that falls to 75% (*p* = 0.0287).

From a structural point of view the analyzed data that gave the best results: spaces provided for staff comfort and breaks (81%), the availability of a shower (94%) and differentiated in/out pathways both for staff and materials (92%) made staff feel safe while performing tasks in the COVID-19 unit. While only a few did not appreciate the implemented measures.

### 3.2. Health Surveillance

The participants were questioned about any possible biological injury that might have occurred in the COVID-19 unit. Four hundred forty two (97%) reported no biological injuries. Four (2%) splashes of biological fluid were reported by nurses. There was no-significance in the stratification by age (*p* = 0.6502) or length of service (*p* = 0.7355).

The hospital health physician reported a 0.012 incidence of biological injuries and 0.078 incidence of staff requests for additional consultations.

No cases of COVID-19 positivity were reported during screening for all the staff working in the COVID-19 area.

Health surveillance with pre-, peri- and post- COVID-19 unit entry, reported significant data, with no cases of COVID-19 positivity.

The health surveillance in place led to acceptance of the model in 112 (83%) versus 13 unsatisfied participants (10%). Greater criticality was once again shown in the younger age group with the lowest satisfaction figure which rose to 21% (*p* = 0.0513).

### 3.3. Training

A total of one hundred fifteen (85%) reported having acquired confidence thanks to the training received, 13 (10%) disagreed, and 7 (5%) reported indifference. A total of six (25%) of the younger respondents disapproved about the adequate level of training (*p* = 0.0036), as did 15% of the newly experienced ICU staff. Equally, staff with less ICU seniority showed less satisfaction with the training received before their entry into the COVID-19 area with a dissatisfaction figure that rises to 15% and satisfaction that falls to 76% (*p* = 0.0446).

**Figure 2:**
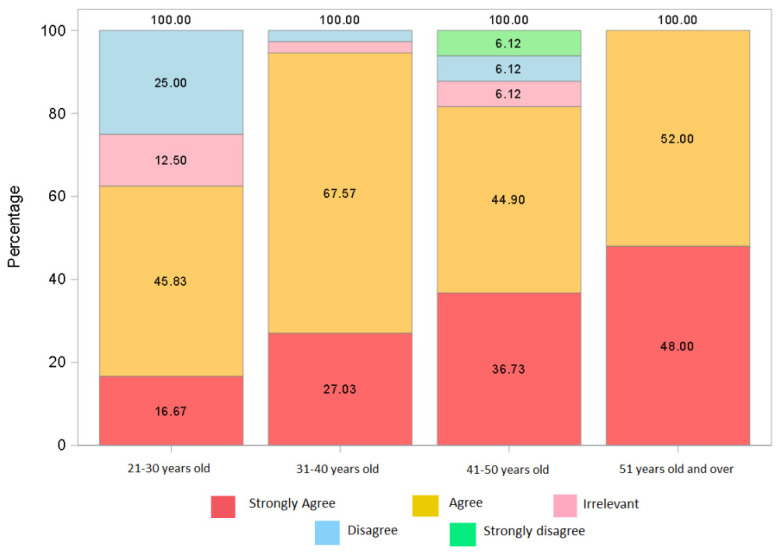
Training stratified by age (Q13)—(Frequency response “Strongly disagree”, “Disagree”, “Irrelevant”, “Agree”, “Strongly agree”; 20–30 years: 0, 6, 3, 11, 4; 31–40 years: 0, 1, 1, 25, 10; 41–50 years: 3, 3, 3, 22, 18; >50: 0, 0, 0, 13, 12).

The respondents were asked if the training received in terms of donning PPE was satisfactory, with positive responses of 112 (83%) versus 11 unsatisfied (8%). Similar to the question on satisfaction with training received, the greatest critical issues were presented by the younger groups of the population with a negative figure of 25% and indifference at 12.5%, a value that progressively improves up to 100% satisfaction among the over 50s (*p* = 0.0003). Figure 2. 

Similarly, the youngest in the COVID-19 ICU in terms of seniority have shown greater dissatisfaction with the training received, with a negative figure of 21% (*p* = 0.0021). Great homogeneity in the results related to the organization that has differentiated the inbound and outbound routes from the COVID-19 area between personnel and materials: welcomed by 124 respondents (92%) without particular distinctions among subgroups.

### 3.4. Staff Risk Perception

A total of seventy six (57%) answered they live with family members belonging to categories at high risk for contracting COVID-19. One hundred fifteen (79%) showed fear of becoming a vehicle towards family members in the transmission of the virus, and 18 (14%) were not afraid. In the stratification by age, the younger age groups showed greater fear of becoming vectors, a fear that seems to reduce with age (*p* = 0.0281). A total of thirty two (42%) did not report an increase in concerns and fear in relation to their professional role (*p* = 0.0264).

The vast majority of respondents perceived COVID-19 as a dangerous and deadly disease (94%) not only for themselves but even more as a vector towards their families (79%). A total of 55% of staff took isolation measures and moved away from their home by changing personal habits.

Homogeneity of data was appreciated in the last two questions of the questionnaire that investigated the perception of COVID-19 as a fatal disease and as a condition that significantly changed personal habits of social life: for both questions the answers were 94% in agreement.

**Figure 3:**
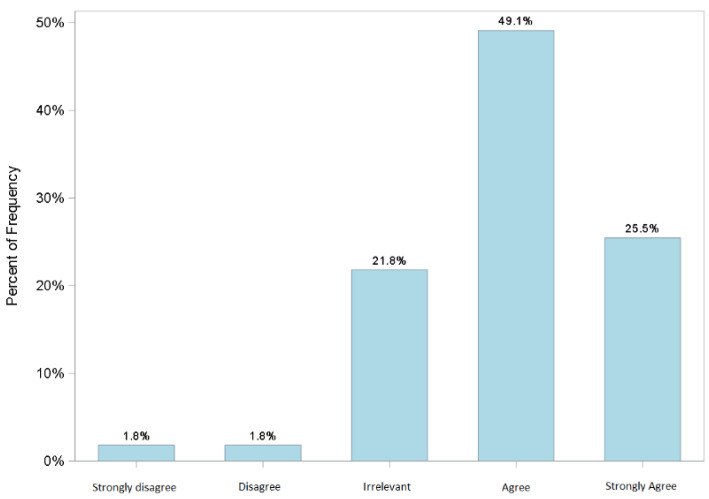
Q23. In the first few days after your access to the COVID-19 area, did you take isolation measures upon returning home to protect your family?

**Figure 4:**
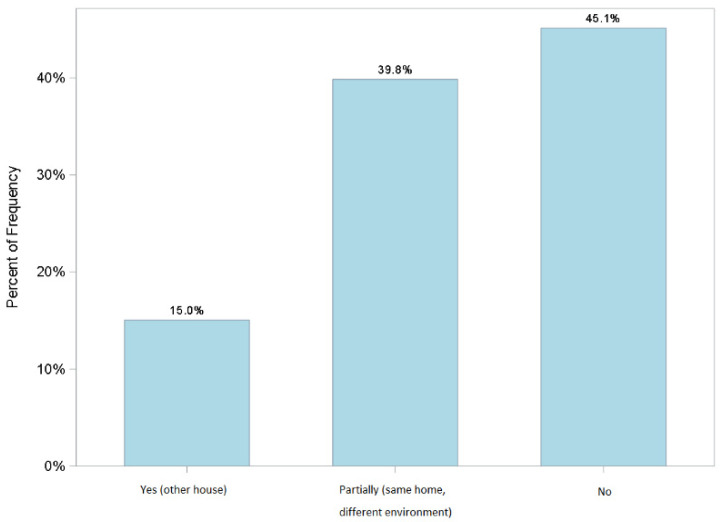
Q25. I returned to normal daily habits because I felt sufficiently reassured by the implementation of the corporate organizational model.

The organizational model was perceived overall as appropriate (91%) to guarantee respondents’ health. After a couple of days of service in the COVID-19 area, 75% of the 55% of the staff who had changed personal habits and had isolated themselves, returned to normal habits and homes (Figure 3 and Figure 4) despite the fact that more than half, (52%) lived with family members considered to be at high risk. This is a significant finding in the study where only two people (3%) of the 55, returned home but did not associate this to the organizational model (dates not statistically significant).

### 3.5. Adherence to the Use of PPE

A total of ninety five percent of respondents said they used PPE before entering the unit. Another four (3%) declared that PPE were not available. In relation to the time spent in the unit, the higher percentage of compliance is represented by respondents who have spent between three, and six or more months in the unit with (78) 58% participants replying “yes”. A total of one hundred twenty seven (94%) respondents were compliant in disinfecting their gloves with antiseptic gel before and after contact with patients, two (1%) declared they have never disinfect their gloves, and six (4%) sometimes. An increase in the attention to disinfection of gloves seems to grow with age with 100% compliance in the over 50 s (*p* = 0.0011).

A total of eighty three percent considered the training received during the dressing and undressing phase to be adequate.

## 4. Discussion

The study highlighted how in general the organizational model adopted was perceived as safe. In addition, the implementation of some innovations in the hospital organization such as the differentiation of inbound and outbound routes between people and materials, the refreshment area and the shower have been highly appreciated and have given added value. On the other hand, while in general the training was perceived as suitable, the greatest dissatisfaction was shown by groups with less experience or age, a warning that necessarily must be addressed in the near future. This survey has several limitations that should be acknowledged. First, the validity of the survey depends on a high response rate among the targeted respondents and we did not receive a high response rate from non-clinical staff. The non-clinical staff are not strictly dependent from the administrative point of view but employed by an external company that provides service in an agreement with the hospital. Therefore, although invited to participate, it was not possible to achieve full participation in the survey. The survey was submitted in summertime; this could have had an impact on the response rate as this period is characterized by holiday leave requests.

Second, the data presented is to be considered the personal opinion of the respondent at the time of the submission, since the survey was submitted close to the closure of the COVID-19 unit, this may not necessarily reflect the actual feedback that it would have been given if the questionnaire was submitted at an early stage of their service in the COVID-19 unit.

The emergency conditions in which the organizational model was adopted did not allow us to investigate the perception of the risks related to the service carried out in the COVID-19 area, and the acceptance of the measures implemented in that period. The survey was in fact sent out at the end of the pandemic wave, just before closing the COVID-19 unit. In light of the subsequent waves, and of a reorganization of ISMETT, a further survey is being prepared and will be submitted to evaluate the acceptance of the measures over time, and the experience value.

However, the study has some strengths: it spotlights the topic of perception of risk among staff during an emergency situation.

It gave a voice to staff about how they felt facing an unplanned emergency situation and explored the perceived risk. It gave feedback to those who implemented the organizational model on the basis of knowledge emerging from the scientific literature allowing them to understand if their work has helped those exposed on the front line.

It is understandable that younger subjects and those with less experience of intensive care units may have suffered more stress that did not allow them to perceive the organizational model adopted as safe.

Studies among health care workers involved in the COVID-19 emergency [15,16,17], highlighted a strong knowledge of the characteristics of the disease and its effects, as well as of the possible treatments. In terms of the perception of the risk, it was emphasized how the use of adequate PPE was perceived as the essential tool for preventing the risk of contracting the infection which, however, was not largely considered fatal. Bhagavathula AS et al., highlighted how the response of the health care workers in terms of maintaining a proper level of attention (48%) was not adequate and consequently there was a high incidence of infections among health care workers. Therefore, a strong knowledge of the disease (over 90%) was matched by an acceptable perception of risk (78%) but with a moderately low compliance with the precautions to be adopted (48%), and a high incidence of positive cases among staff. Riccò M et al., in the early stages of the pandemic analyzed the perception of infectious risk among Italian health care workers, and highlighted how the disease was perceived as a normal seasonal flu, and how the use of PPE was not considered essential at that time [18]. The results of this study cannot therefore be considered significant due to when the study was conducted. Finally, the study analyzed the perception of the authority of institutions and discordances between scientific experts. With regard to the psychological impact of COVID-19 among health care workers: stress (14%), depression (12%) and anxiety (31%) were the main findings. The greatest incidence of psychological impacts of COVID-19 among health care workers were found among nurses and radiology technicians, in health care workers who had experienced a death of a family member, or health care workers cohabiting with COVID-19 infected persons (Erquicia, et al., 2020 [19]).

The time that elapsed from the onset of the global health problem to the request from the Italian NHS to open a COVID-19 ICU area, short as it was, allowed us to acquire the necessary data from our other experiences to implement a secure model for staff: this survey certifies the effectiveness of the work done from an organizational point of view.

## 5. Conclusions

The perception of risk among health care providers is the result of multiple factors ranging from perceived health risk to social risk (health risk for family members and stigma) and accepting risk related to one’s profession.

The vast majority of respondents perceived the overall model applied during an unexpected and emergency situation as appropriate.

## Figures and Tables

**Table 1:** Implemented measures.

Structural and Training Procedural Elements Implemented for the Opening of the COVID-19 ICU Area	
Structural	Differentiated inbound and outbound routes from the COVID-19 area between personnel and materials.
	Inbound and outbound filter areas.
	Refreshment area for staff.
	Implementation of shower in the changing room leaving the COVID-19 area.
	Negative pressure rooms/outpatient room to safely manage suspected patients.
	Triage point at the entrance of the hospital with thermoscanner.
	Personal and general protective equipment availability/hand sanitizer dispensers.
Education	Hand hygiene.
	Donning and doffing personal protective equipment.
	Education on differentiated inbound and outbound routes.
	Education on medical waste management, supplies and transport of biological samples.
	Theoretical and practical training for 23 nurses with no experience in critical nursing (2557 h).
	Experienced critical care nurse educators (2) assigned to the COVID-19 ICU.
Policies and procedures	Infection control policies and procedures implementation.
Health surveillance	Evaluation by the occupational physician with the release of eligibility opinion for possible assignment to the COVID-19 ICU.
	Serological tests, rapid or molecular antigenic swabs.
	Molecular swab every 14 days for dedicated COVID-19 staff.
	Molecular swab a week after completion of the service in the COVID-19 area.
General	Modified working shifts.
	Newly implemented work organizational briefing.

**Table 2:** Questionnaire and results.

General Data		Frequency	Percent
Q1. Age.	20–30 years	27	17.65
	31–40 years	40	26.14
	41–50 years	55	35.95
	over 50 years	31	20.26
Q2. Sex.	M	86	56.21
	F	67	43.79
Q3. Work/seniority in ICU settings.	<6 months	40	26.14
	6 months–1 year	16	10.46
	1–3 years	6	3.92
	>3 years	91	59.48
Q4. Professional role.	Nurse	74	48.37
	Physician	12	7.84
	Physiotherapist	11	7.19
	Aide	35	22.88
	Radiology technician	5	3.27
	Housekeeping	3	1.96
	Other	13	8.50
Q5. How long have you been working in the COVID-19 area?	<1 month	42	27.45
	1–3 months	28	18.30
	3–6 months	35	22.88
	>6 months	48	31.37
Clinical care tasks carried out on COVID-19 patients			
Q6. Did you provide direct care to confirmed COVID-19 patients (e.g., CPR, intubation, extubation, circuit change, aerosol therapy, tracheotomy, bronchoscopy, patient hygiene, mobilization)?	Yes.	98	67.12
	No.	48	32.88
Q7. Did you have direct contact with the environment where confirmed COVID-19 patients were treated (e.g., bed, linen, medical equipment, restrooms, environmental hygiene)?	Yes.	113	77.40
	No.	33	22.60
Q8. During the activity in the COVID-19 area did you suffer any biological injury?	Yes, splashes of biological fluid/respiratory secretions in my mouth/nose mucosa.	2	1.37
	Yes, splashes of biological fluid/respiratory secretions on intact skin.	2	1.37
	Yes, puncture/cut with material contaminated by biological fluid/respiratory secretions.	0	0
	No injury.	142	97.26
Preventive measures implemented by the corporate organization			
Q9. The organizational model adopted at ISMETT (e.g., dressing and undressing area, cafeteria, changing rooms and shower, training, health surveillance, in/out routes) made me feel safe while working in the COVID-19 ICU.	Strongly disagree	0	0
	Disagree	13	9.63
	Irrelevant	9	6.67
	Agree	58	42.96
	Strongly agree	55	40.74
Q10. Before entering the COVID-19 ICU, did you wear personal protection equipment (PPE) according to the corporate policies?	Yes.	128	94.81
	I don’t think all PPEs are necessary.	0	0
	Not all PPEs are always available.	4	2.96
	Other (specify).	3	2.22
Q11. Before and after contact with the patient or entering/leaving the room, did you disinfect the gloves with antiseptic gel?	Never	2	1.48
	Sometimes	0	0
	Almost always	6	4.44
	Always	127	94.07
Q12. Before and after contact with the surrounding environment (e.g., bed, door handle, infusion pump, ventilator, ECMO, monitor) of with a COVID-19 patient, did you disinfect the gloves with antiseptic gel?	Never	3	2.22
	Sometimes	1	0.74
	Almost always	12	8.89
	Always	119	88.15
Q13. The training and in-services (e.g., dressing/undressing, use of PPEs during shifts, work organization) made me confident to perform my tasks in the COVID-19 ICU.	Strongly disagree	3	2.22
	Disagree	10	7.41
	Irrelevant	7	5.19
	Agree	71	52.59
	Strongly agree	44	32.59
Q14. I received sufficient training/in-services on proper hand hygiene procedure.	Strongly disagree	0	0
	Disagree	3	2.22
	Irrelevant	6	4.44
	Agree	53	39.26
	Strongly agree	73	54.07
Q15. I received sufficient training on the correct sequence of COVID-19 dressing and undressing.	Strongly disagree	2	1.48
	Disagree	9	6.67
	Irrelevant	12	8.89
	Agree	65	48.15
	Strongly agree	47	34.81
Q16. The differentiated in/out pathways, both for staff and materials, made me feel safe while performing my tasks in the COVID-19 ICU.	Strongly disagree	0	0
	Disagree	4	2.96
	Irrelevant	7	5.19
	Agree	65	48.15
	Strongly agree	59	43.70
Q17. The health surveillance system made me feel safe during my work in the COVID-19 ICU.	Strongly disagree	2	1.48
	Disagree	11	8.15
	Irrelevant	10	7.41
	Agree	64	47.41
	Strongly agree	48	35.56
Q18. The areas provided for staff comfort and breaks made me feel safer working in the COVID-19 ICU.	Strongly disagree	1	0.74
	Disagree	8	5.93
	Irrelevant	17	12.59
	Agree	60	44.44
	Strongly agree	49	36.30
Q19. I believe taking a shower outside the COVID-19 area is an additional safety tool for health care providers.	Strongly disagree	0	0
	Disagree	1	0.74
	Irrelevant	7	5.19
	Agree	41	30.37
	Strongly agree	86	63.70
Staff risk perception			
Q20. Composition of your family.	I live alone.	19	14.29
	I live with children younger than 18 years of age.	50	37.59
	I live with people at greater COVID-19 risk (e.g., elderly, fragile).	26	19.55
	I live with people not included in the categories at risk.	38	28.57
Q21. I’m afraid to transmit COVID-19 to my family or loved ones when I return home from work in the COVID-19 area.	Strongly disagree	7	5.26
	Disagree	11	8.27
	Irrelevant	10	7.52
	Agree	67	50.38
	Strongly agree	38	28.57
Q22. I consider myself among those at higher risk of contracting COVID-19 infection due to my activity in the COVID-19 ICU.	Strongly disagree	15	11.28
	Disagree	27	20.30
	Irrelevant	22	16.54
	Agree	50	37.59
	Strongly agree	19	14.29
Q23. In the first few days after your access to the COVID-19 area, did you take isolation measures upon returning home to protect your family?	Yes (another house).	20	15.04
	Partially (same home, different environment).	53	39.85
	No.	60	45.11
Q24. Did you return to your normal daily habits after a few days?	Yes	55	75.34
	No	18	24.66
Q25. I returned to normal daily habits because I felt sufficiently reassured by the implementation of the corporate organizational model.	Strongly disagree	1	1.82
	Disagree	1	1.82
	Irrelevant	12	21.82
	Agree	27	49.09
	Strongly agree	14	25.45
Q26. I think COVID-19 is a dangerous and deadly disease.	Strongly disagree	0	0
	Disagree	4	3.01
	Irrelevant	3	2.26
	Agree	61	45.86
	Strongly agree	65	48.87
Q27. COVID-19 changed my daily life habits (physical distancing, frequency of family meetings, social contacts).	Strongly disagree	2	1.50
	Disagree	3	2.26
	Irrelevant	3	2.26
	Agree	57	42.86
	Strongly agree	68	51.13

## Data Availability

Data will be available upon request.
